# Right subclavian vein catheterism complication due to a 'foreign body': a case report

**DOI:** 10.1186/1752-1947-4-327

**Published:** 2010-10-19

**Authors:** Zacharoula Sidiropoulou, Pedro João, Paula Vasconcelos, Cristiana Couceiro

**Affiliations:** 1 Surgery Department, Hospital N. S. Rosário, Av. Forças Armadas, Barreiro, Portugal; 2 Radiology Department, Hospital N. S. Rosário, Av. Forças Armadas, Barreiro, Portugal

## Abstract

**Introduction:**

Central venous access devices are widely used in hospital practice. Complications associated with their use are well described and reviewed. In this paper, we report a former complication that in turn created a new complication during a standardized procedure.

**Case presentation:**

We report the case of an 81-year-old Caucasian woman requiring total parenteral nutrition due to a high-debt enterocutaneous fistula. In a previous right subclavian catheterization a fragmentation of the tip of the catheter, probably not recognized at the time, provoked an extrinsic compression of the vessel.

**Conclusion:**

Fragmentation of a central venous catheter is a possible complication of catheterization and can be missed. Control of a catheter is imperative after its removal, even if not always practiced.

## Introduction

Central venous access devices are widely used for the administration of antibiotics and chemotherapeutic drugs, total parenteral nutrition, providing high-flow access for hemodialysis and plasmapheresis, and central venous pressure monitoring. In many cases the same patient will undergo this procedure on more than one occasion, leading to an increase in the possibility of complications.

Central venous catheterization has multiple advantages, for example the reduction of irritation and thrombosis of smaller peripheral veins, the avoidance of peripheral phlebitis and scarring, and a much better patient tolerance. The immediate complications are insertion site bleeding, pneumothorax and hemothorax, arterial puncture, displacement of the catheter and fragmentation of the catheter [1,2]. Late complications can be catheter infection, surgical site infection, occlusion, endocarditis, and valve embolism [3].

## Case presentation

An 81-year-old Portuguese Caucasian woman, in the immediate post-operative period for incisional relapsing hernia, developed an enterocutaneous, high debit fistula requiring total parenteral nutrition.

Our patient presented no other major medical comorbidity. Her surgical history consisted of seven abdominal operations. The first was a total hysterectomy with bilateral adnexectomy by a midline abdominal incision (1979). Secondly, two years later she developed an incisional hernia and was submitted to a herniorraphy (1981). Her third operation was a laparotomy for intestinal occlusion due to adhesions (1984). Then, three years later she developed a new incisional hernia that was corrected by hernioplasty (1991). Because of surgical site infection, the prosthesis had to be removed and replaced (1991). After a new episode of intestinal occlusion, this time with necrosis, a segmental resection of ileum (2004) was performed, after which she presented with a recurrence of the incisional hernia and was operated on again by hernioplasty (2008). There was a new recurrence of the incisional hernia one year later and an application of biological prosthesis was completed (2009).

The surgical team had no data about the intestinal occlusions and the following resection operations that were reported later by one of the daughters of our patient, who lived abroad. No detailed medical reports had been presented, and it seemed that some of the operations had been performed in another hospital during occasional stays of our patient at her daughter's home abroad.

The first approach to the central venous catheterization was made through the right subclavian vein, following the Seldinger technique. During the introduction of the wire, resistance was encountered, so the surgeon extracted the guide and reattempted introduction. During this second attempt the arterial vessel was accidentally punctured and local compression was applied to successfully stop the bleeding.

A new approach was attempted with the same catheter on the left subclavian vein, which was successful and without complications. A control chest X-ray was ordered and showed the correct positioning of the catheter.

**Figure 1:**
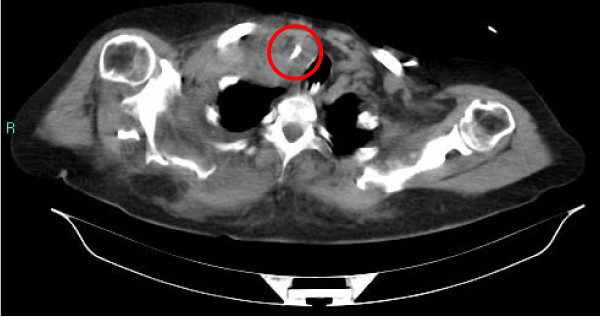
**Previous catheter tip**.

**Figure 2:**
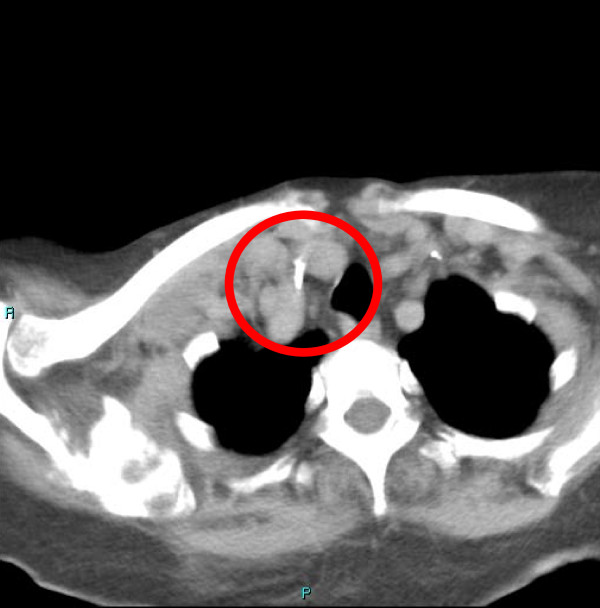
**Previous catheter tip localization between the two right subclavian vessels**.

**Figure 3:**
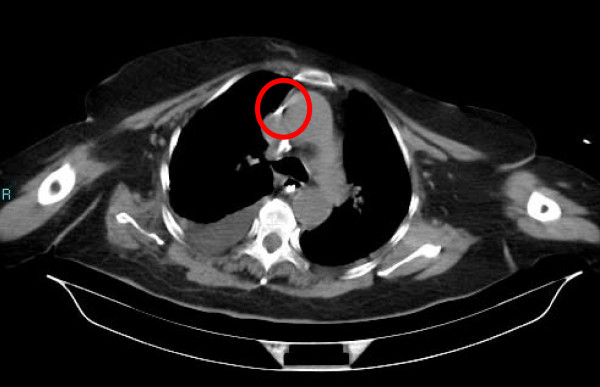
**Left subclavian catheter tip**.

The following day, during a medical review, a hematoma on the neck of our patient located at the right supraclavicular fossa was noted, so a computed tomography (CT) contrast scan was performed. The results of the CT scan showed a small right supraclavicular fossa hematoma, with no active bleeding, and a triangular foreign body of metallic tomographic appearance, approximately 5 mm in length, in the interstitial space between right subclavian vein and artery. There was no pneumothorax or hemothorax. The left subclavian vein catheter was intact and well positioned (Figures 1, 2, 3).

We presumed that the foreign body detected was a central venous catheter tip. This probably fragmented during the extraction of the device placed in 2004 when our patient underwent intestinal resection, and later migrated to the interstitial space between the vessels. However, we have no means to confirm our theory. The only certainty we had is that it is not a complication of our procedure since we did not change the catheter during its replacement and our patient's CT scan results showed two catheter tips.

## Discussion

We did not take any additional surgical or interventional measures, since our patient was asymptomatic and the 'embolus' was fixed in the interstitial space. Nevertheless, our patient has continued taking enoxaparin 20 mg subcutaneous daily in order to prevent any thromboembolic complications [4].

## Conclusions

Taking of a thorough medical history is extremely important for the safe and successful management of a patient, but it is not always possible to obtain. Central venous catheterization complications can be misdiagnosed by the time they occur. When rare difficulties during catheter placement occur, the possibility that relevant data could be missing from a patient's clinical history with regard to previous complications should be considered. It is also good practice to check catheters and perform microbiological cultures of the tip. An ultrasound-guided catheter insertion could possibly have detected, in real time, the extrinsic compression and further manipulation could have been avoided.

## Competing interests

The authors declare that they have no competing interests.

## Authors' contributions

ZS analyzed and interpreted data from our patient regarding the surgical procedure. PJ, PV and CC performed the radiological tests and oriented their interpretation. All authors read and approved the final manuscript.

## Consent

Written informed consent was obtained from the patient for publication of this case report and any accompanying images. A copy of the written consent is available for review by the Editor-in-Chief of this journal.
